# From Heritage to Global Presence: The Case for a PubMed-Indexed Greek Surgical Journal

**DOI:** 10.7759/cureus.91668

**Published:** 2025-09-05

**Authors:** Dimitrios Moris, Emmanouil Giorgakis

**Affiliations:** 1 Surgery, MedStar Georgetown University Hospital, Washington, D.C., USA; 2 Surgery, University of North Carolina at Chapel Hill, Chapel Hill, USA

**Keywords:** clinical research, greece, journal, pubmed database, type of surgery

## Abstract

Despite Greece’s rich surgical heritage and internationally recognized clinicians, the country lacks a PubMed-indexed surgical journal. This gap reflects systemic barriers, including limited funding for academic publishing, the absence of a sustained editorial infrastructure, insufficient English-language output, and low prioritization of journal development in academic policy. Establishing such a platform would enhance Greek surgical research, promote international collaborations, and enhance academic visibility. The creation of a PubMed-indexed Greek surgical journal requires a strategic plan: unification of pre-existing journals, securing institutional and society support, implementing rigorous peer review, publishing in English, and pursuing indexing through MEDLINE and Scopus. A committed editorial board, robust digital presence, and integration into global networks could transform Greece’s surgical scholarship from a largely local discourse into a global contributor. Addressing these challenges is essential for ensuring that Greece’s surgical voice is heard and recognized internationally.

## Editorial

Introduction

From the Hippocratic tradition to pioneering advances in modern clinical surgery, Greece has made extensive contributions to the foundations of medicine [1]. Greek surgeons have led internationally recognized programs, produced highly cited research, and held leadership positions in global surgical societies. However, despite this strong academic and clinical footprint, Greece does not currently have a PubMed-indexed national surgery journal. This absence represents more than a symbolic gap; it limits the international visibility of Greek research, diminishes opportunities for young surgeons to publish in a nationally affiliated journal recognized abroad, and weakens the country’s capacity to shape the global surgical discourse.

Barriers to indexing

The absence of such a journal is not due to a lack of talent or research interest, but rather to a series of persistent structural challenges. Publishing efforts in Greek surgery are highly fragmented, with multiple society journals operating independently, often with limited scope, small circulation, and a low volume of original research submissions [2]. High-quality surgical work from Greece is often directed to established international journals with higher impact factors and broader readership, leaving domestic journals with fewer competitive manuscripts [3].

In addition, many Greek surgical journals struggle with the infrastructural demands of PubMed indexing. Maintaining a consistent publication schedule, robust peer-review processes, and professional editing requires sustained financial support and experienced editorial management resources that are often scarce [4]. The language of publication is another limiting factor. Although English dominates scientific publishing worldwide [5], many Greek journals continue to publish in Greek or in bilingual formats, which limits international reach and citation potential [6].

Finally, there has been no coordinated national strategy to establish a flagship surgical journal [7]. Unlike countries of comparable or smaller size, such as Portugal, Norway, and New Zealand, that have successfully developed PubMed-indexed national journals through collaboration between societies, universities, and government agencies, Greek efforts have remained decentralized and unaligned.

Why it matters

The lack of a PubMed-indexed Greek surgical journal has tangible consequences. Without a central platform for dissemination, Greek surgical research is dispersed across foreign publications, reducing its visibility as a collective national output. Young surgeons lose the opportunity to publish in a high-quality, nationally affiliated journal that is recognized in international databases. This gap also limits Greece’s ability to consolidate its academic voice, influence surgical practice guidelines, and highlight innovations emerging from its institutions. In contrast, other countries have used national journals as both an academic showcase and a training ground for future leaders in surgical research and publishing [7,8].

A roadmap forward

Overcoming these barriers will require coordinated action from the surgical community. The first step is the unification of existing society journals into a single, multidisciplinary surgical publication capable of attracting high-quality submissions from across the country. Commitment to English-language publishing, perhaps accompanied by Greek-language abstracts to maintain local accessibility, is essential for indexing and international readership.

An inclusive editorial board composed of leading Greek surgeons from various institutions, members of the Greek surgical diaspora, and respected international experts would enhance credibility and attract diverse submissions [9]. Sustainable funding models, drawing on society memberships, academic institutions, and ministry-level grants, are needed to maintain consistent editorial quality and publication schedules [10].

Several funding models could be pursued: (1) Society-based support: The Hellenic Surgical Society and subspecialty societies could allocate a portion of annual membership fees to cover editorial costs, similar to models used by European societies. (2) University and hospital contributions: Academic departments could commit modest yearly funding in exchange for institutional representation on the editorial board and publication discounts for their faculty. (3) Diaspora philanthropy: Greek surgeons abroad, many of whom are already benefactors of academic initiatives, could provide start-up endowments or sponsor named editorial positions. (4) Government and foundation grants: The Ministry of Health, Ministry of Education, and organizations such as the Stavros Niarchos Foundation could support the journal as part of a broader national strategy to elevate Greek science internationally. (5) Hybrid open-access model: To minimize financial barriers for authors, a mixed system of low article processing charges for funded researchers, waivers for trainees, and institutional underwriting could balance accessibility with sustainability. Partnerships with established international publishers may further reduce costs by providing digital platforms and distribution infrastructure.

In this context, the Greek surgical diaspora emerges as a uniquely powerful asset. Greek surgeons working in the United States, the United Kingdom, Germany, and other centers of academic excellence hold leadership roles in universities, societies, and international editorial boards. Their experience can be directly leveraged to guide the creation of a PubMed-indexed Greek surgical journal. Diaspora faculty can contribute as senior editors, peer reviewers, and mentors, ensuring that domestic authors receive constructive feedback aligned with the standards of globally recognized journals. Importantly, their involvement would also help establish trust with indexing bodies such as PubMed and Scopus, which look for editorial boards with international credibility.

Beyond mentorship, diaspora networks can serve as conduits for international collaborations. Many multicenter studies on hepatopancreatobiliary surgery, transplantation, and minimally invasive techniques already involve Greek investigators abroad. By channeling such collaborations through a national journal, Greece could position its publication as a hub for cross-border research. For example, joint position papers, consensus guidelines, and surgical education initiatives developed between domestic and diaspora surgeons could both elevate the journal’s content and ensure citation traction internationally. This model has proven successful in other countries with vibrant diasporas, where national journals became platforms for bridging local practice with international standards.

Mentorship is particularly crucial for early-career Greek faculty. Many young surgeons are skilled clinicians, but they often lack systematic training in academic writing and publishing. Through structured diaspora engagement (virtual workshops, co-authorship opportunities, and editorial shadowing programs), junior faculty can gain invaluable skills that would otherwise require years of immersion abroad. This mentorship pipeline would not only increase submission quality but also foster a new generation of academically oriented Greek surgeons committed to sustaining the journal long-term.

Finally, proactive solicitation of manuscripts from multicenter Greek studies, national surgical guidelines, and collaborations with diaspora academics could ensure a steady stream of impactful content. Recognition of publications in such a journal for academic promotion and grant evaluation would further incentivize submissions [11].

Conclusions

The creation of a PubMed-indexed Greek surgical journal is both feasible and urgently needed. Such a platform would unify the national academic voice, improve the visibility of Greek research, and inspire younger generations to contribute to the literature. Moving from heritage to global presence requires unity, vision, and investment, but the potential benefits to Greek surgery, both nationally and internationally, are substantial and lasting.
